# LEPRE1 shapes a non‐flamed tumour immune landscape and predicts the prognosis in esophageal squamous cell carcinoma

**DOI:** 10.1002/ctm2.1473

**Published:** 2023-11-23

**Authors:** Meng Liu, Yu Ma, Yunquan Guo, Yue Ma, Xinxin Bao, Xiaomei Lu

**Affiliations:** ^1^ State Key Laboratory of Pathogenesis, Prevention and Treatment of High Incidence Diseases in Central Asia Affiliated Tumor Hospital of Xinjiang Medical University, Xinjiang Uygur Autonomous Region Urumqi PR China; ^2^ The Clinical Medical Research Center of Breast and Thyroid Tumor in Xinjiang,Xinjiang Uygur Autonomous Region Urumqi PR China; ^3^ Department of Clinical Laboratory The Fourth People’ Hospital of Urumqi, Xinjiang Uygur Autonomous Region Urumqi PR China; ^4^ Department of Pharmacy Xinjiang Medical University, Xinjiang Uygur Autonomous Region Urumqi PR China; ^5^ Clinical Medical Research Institute, First Affiliated Hospital of Xinjiang Medical University, Xinjiang Uygur Autonomous Region Urumqi PR China

Dear Editor,

Esophageal cancer contributes to over 400 000 deaths around the world each year.^1^, ^2^ Recently, immune checkpoint blockade (ICB) has been used in esophageal squamous cell carcinoma (ESCC),^3^ but ICB demonstrated low overall response rates and acquired resistance in patients.^4^ Therefore, finding a suitable target that predicts the prognosis in ESCC for combinatorial therapy with ICB is urgently needed.

In our study, Leucine proline‐enriched proteoglycan 1 (LEPRE1), a member of the collagen prolyl hydroxylase family, was identified from our ESCC cohorts (Figure 1A) by differential expression, Log‐rank, cox regression and immunohistochemistry (IHC) in ESCC, which was validated in 45 samples (Figure S1B). Research results suggested significant overexpression of LEPRE1 in ESCC (Figure S1C‐K) and in the cancer genome atlas (TCGA) (Figure S1A). Besides, we found that 71.8% ESCC samples had higher LEPRE1 protein expression than the normal tissues (Figure S1L) by IHC staining in 80 ESCC samples.

**FIGURE 1:**
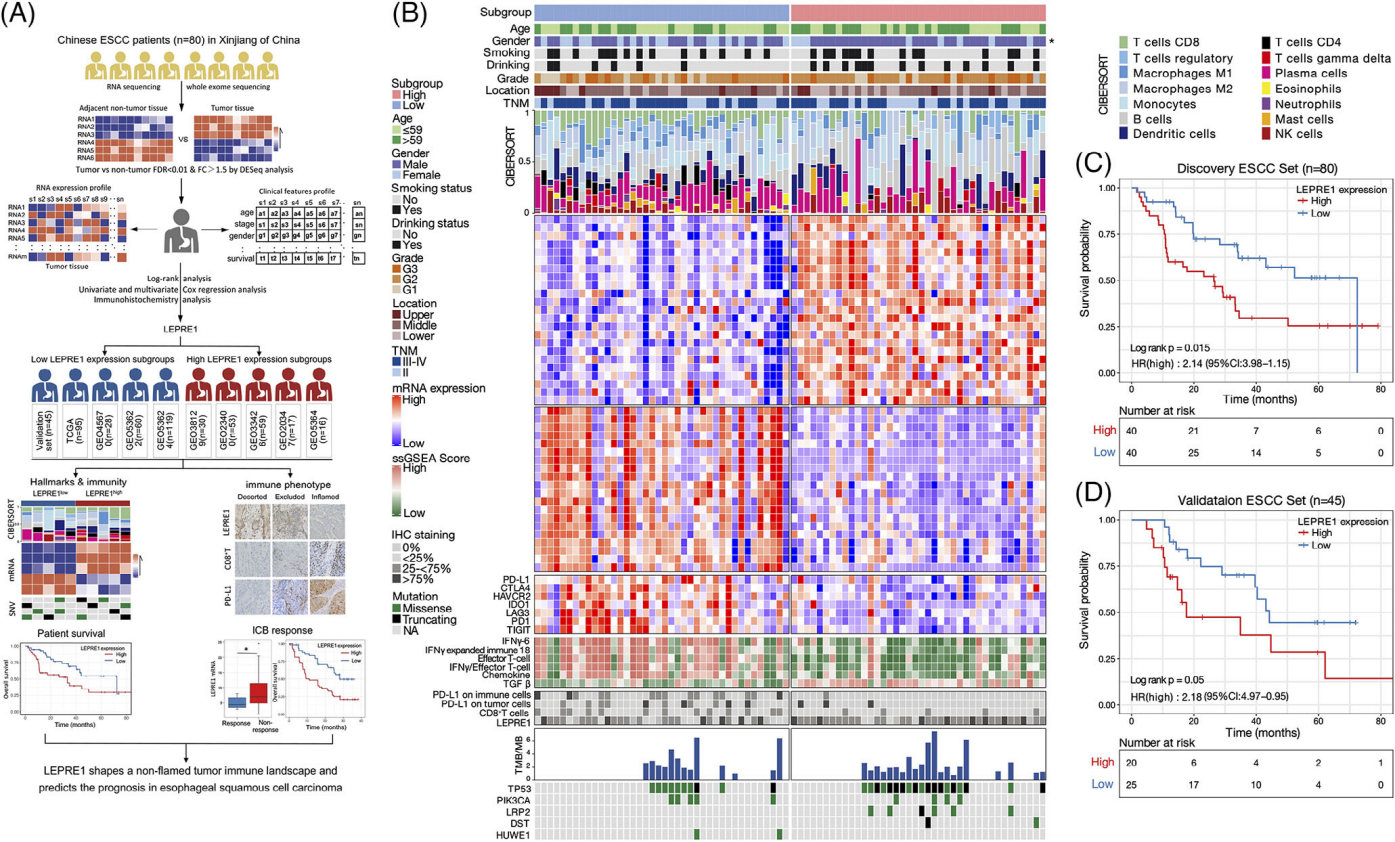
Tumour immune landscape and molecular characteristics between high‐ and low‐LEPRE1 subgroups in esophageal squamous cell carcinoma (ESCC). (A) Overview of the study design. RNA expression profile and WES data of Chinese ESCC samples (*n* = 80) of Xinjiang Tumor Hospital in China were used to detect LEPRE1 expression and validation in Chinese ESCC samples (*n* = 45) and 9 publicly available ESCC cohort. (B) Heatmap representation of the relative mRNA abundance of differentially expressed mRNA and tumour immune landscape between high‐ and low‐LEPRE1 subgroups in our Chinese ESCC cohort (*n* = 80). From top to bottom: clinicopathologic characteristics; immune cell deconvolution by CIBERSORT; differential expression genes; expression of immune checkpoint blockade (ICB); immune therapy response gene sets score; immunohistochemistry (IHC) of LEPRE1, PD‐L1 and CD8^+^T cells at the ESCC tumour; mutation genes. (C and D) Kaplan–Meier curves of overall survival for high‐ and low‐LEPRE1 subgroups in Chinese ESCC set (*n* = 80) and our validation ESCC set (*n* = 45). *p*‐Values were calculated by log‐rank test.

Our study revealed the tumour immune landscape of LEPRE1 in ESCC. First, the low‐LEPRE1 subgroup had a larger proportion of CD8^+^ T cells, Tregs and M1 macrophages by CIBERSORT analysis (Table S1.1). Further, the percentage of CD8^+^T cells estimated by IHC was also higher in low‐LPERE1 subgroup (Table S2.1). Secondly, the study showed that 23 upregulated and 20 downregulated genes were associated with LEPRE1 expression (Figure 1B, Table S1.2). Up‐regulated genes were enriched in epithelial‐mesenchymal transition (EMT), extracellular matrix (ECM) and TGFβ pathway, which were also enriched in tumour samples compared with non‐tumour samples (Figure S2A, Table S1.3). These results suggested that the activity of these pathways was progressively strengthened from non‐tumour to low‐ to high‐LEPRE1 subgroup. Besides, we observed higher expression levels of PD‐1/L1, CTLA4 in low‐LEPRE1 subgroup (Figure 1B, Table S1.4). The presence of PD‐L1 on immune/tumour cells determined by IHC was also higher in low‐LEPRE1 subgroup (Table S2.1).

To demonstrate the function of LEPRE1 on predicting the clinical response of ICB, ssGSEA scores of a 6/18‐gene IFNγ,^5^ an effector T cell,^6^ a combined IFNγ/effector T cell^7^ and a chemokine signature^8^ were higher in low‐LEPRE1 subgroup. While a TGFβ signature^9^ was lower in low‐LEPRE1 (Figure 1B, Table S2.2).

High‐LEPRE1 subgroup had LRP2 and Dystonin (DST) mutations (*p* = 0.037, 0.06); was enriched for TNM III ESCC; and had worse overall survival. While low‐LEPRE1 subgroup had HUWE1 mutation (*p* = 0.05), it was enriched in female patients (*p* = 0.03) with favourable overall survival (Figure 1B). Besides high tumor mutational burden (TMB) was found in low‐LEPRE1 subgroup. These results together showed that immunotherapy could be used in low‐LEPRE1 patients.

To explain the prognostic significance of LEPRE1 in ESCC. A Kaplan–Meier plot demonstrated a separation in the overall survival (OS) rate between high‐ and low‐LEPRE1 patients (Figure 1C). Furthermore, high‐LEPRE1 subgroup had 2.1, 2.14 times higher risks on OS (*p* = 0.017, 0.016) in the univariable and multivariable model after adjusting for the impacts of age, gender, smoking and drinking status and tumour node metastasis (TNM) stage in 80 patients (Table S3). In addition, these clinical results were confirmed with validation (*n* = 45) (Figure 1D) and external ESCC patients (*n* = 119).

According to the results of KEGG and GO enrichment in 80 ESCC samples (Figure S3A‐D), individuals with high‐LEPRE1 had enrichment in the EMT, TGFβ and ECM pathways in TCGA tumour samples (Figure 2A, Table S4). Besides, we identified gene set enrichment analysis (GSEA) enrichment in 80 ESCC samples similar to those found in TCGA samples (Figure 2B,C; Figure S3E,F). We used shRNAs to inhibit the expression of LEPRE1 in KYSE140 and KYSE150 cells in vitro (Figure S3G, H). Cell proliferation, migration and invasion were inhibited upon LEPRE1 mRNA knockdown (Figure 2D,E; Figure S3I,J). By using western‐blot, we discovered that knockdown resulted in enhanced E‐cadherin levels and decreased N‐cadherin and vimentin levels (Figure 2F).

**FIGURE 2:**
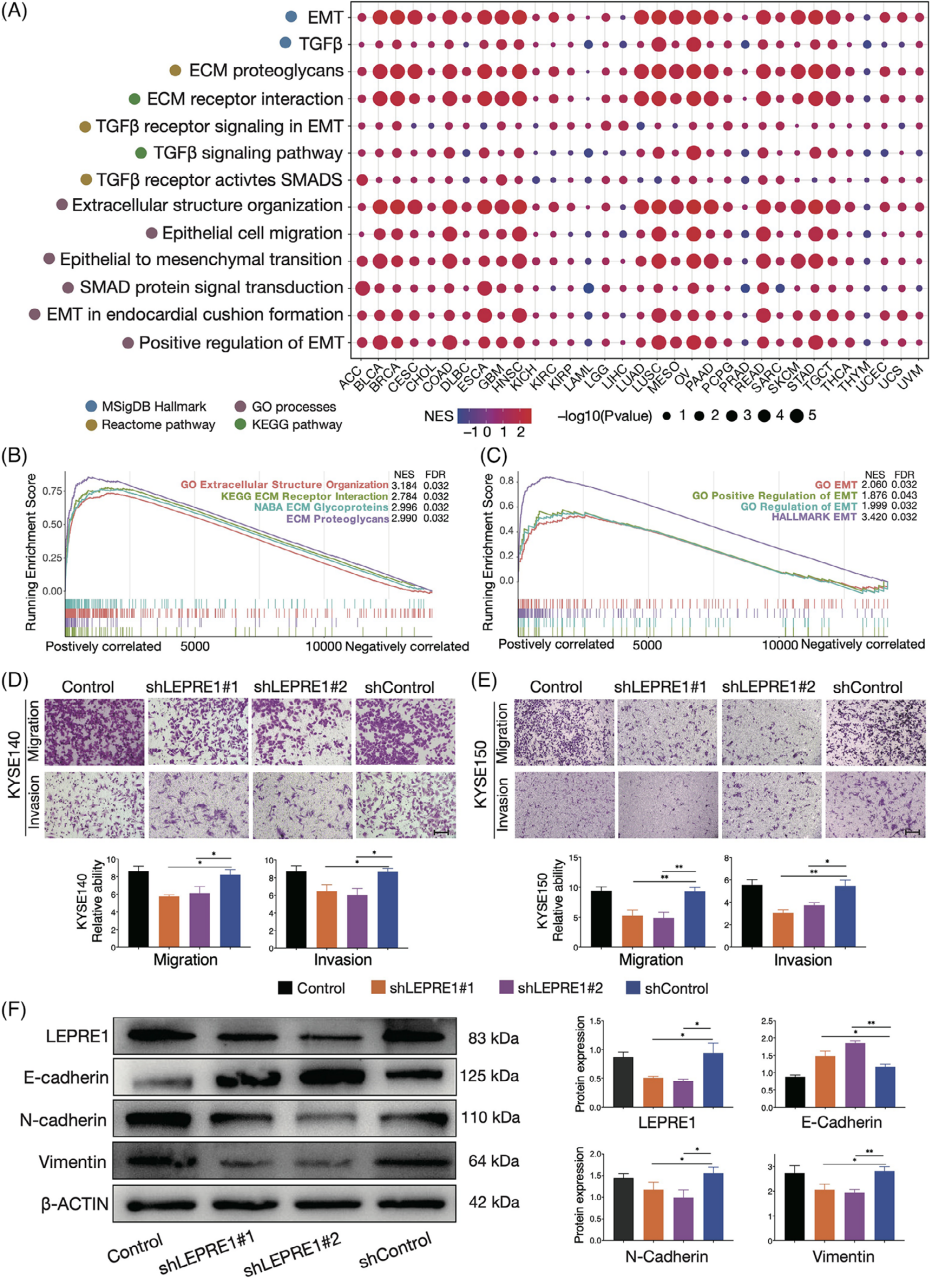
Functions of LEPRE1 on cancer metastasis in esophageal squamous cell carcinoma (ESCC). (A) Pathway enrichment score between high‐ and low‐LEPRE1 subgroups across cancer types in TCGA using GSEA method. Red circles show positively normalized enrichment score (NES), and blue circles show negatively NES. Circles size represent –log10 (*p*‐value). (B and C) GSEA showing significant enrichment of EMT and ECM‐related pathways in the high‐LEPRE1 ESCC samples. (D and E) Silencing the expression of LEPRE1 significantly suppressed KYSE140 and KYSE150 cell migration and invasion. The scale bars correspond to 200 μm. Up panel shows cell migration and invasion images and below panel shows quantification statistics. (F) Western blot analysis of LEPRE1, E‐cadherin, N‐cadherin and Vimentin expression; β‐actin was used as loading control. The plots at the right show the quantification of the intensity of LEPRE1, E‐cadherin, N‐cadherin or vimentin. Error bars denote the standard deviation. Data represent mean ± standard error of the mean (SEM) from three independent experiments. *p < 0.05; **p < 0.01.

**FIGURE 3:**
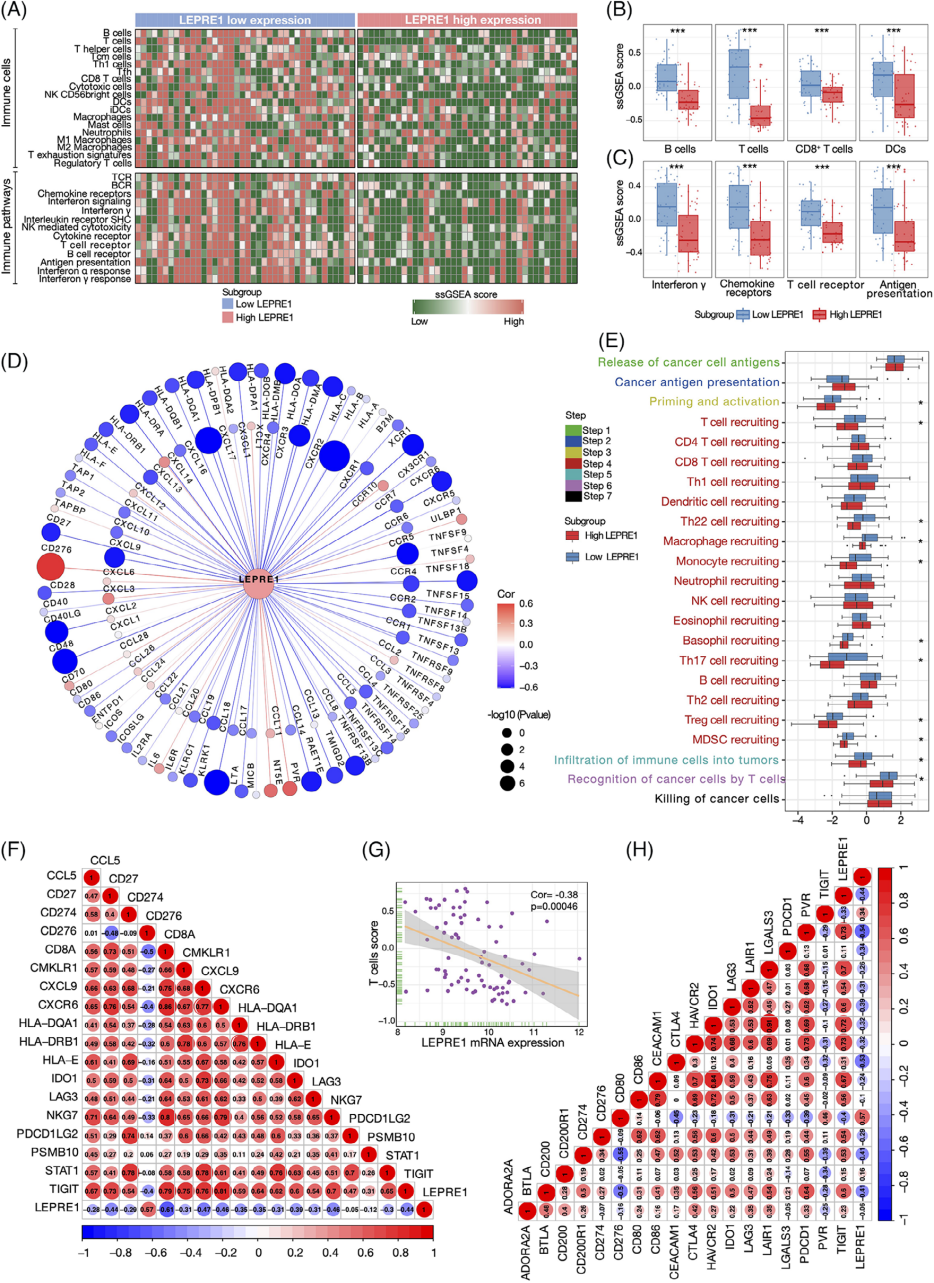
LEPRE1 shapes a non‐inflamed tumour immune landscape in esophageal squamous cell carcinoma (ESCC). (A) Heatmap representation of the infiltration levels of tumour‐associated immune cells, as well as the enrichment score of immune‐related pathways between high‐ and low‐LEPRE1 subgroups in Chinese ESCC cohort (*n* = 80). (B and C) Quantification analyses of immune cells including B cells, T cells, CD8/4^+^ T cells, DCs and immune‐related pathways including interferon γ, chemokine receptors, T cell receptor, antigen presentation in Chinese ESCC. (D) Correlation between LEPRE1 and 122 immunomodulators (chemokine, receptor, MHC and immunostimulator) in Chinese ESCC. (E) Differences in the steps of the cancer immunity cycle between high‐ and low‐LEPRE1 subgroups in Chinese ESCC. (F and G) Correlations among LEPRE1 and critical marker genes of T cells, the T cells score in Chinese ESCC. (H) Correlations between LEPRE1 and immune checkpoint blockade in Chinese ESCC. *p < 0.05; **p < 0.01; ***p < 0.001.

LEPRE1 shapes a non‐inflamed tumour immune landscape in ESCC. Low‐LEPRE1 patients had a higher infiltration levels of CD8^+^ T cells, B cells and T cells (Figure 3A, Table S5.1). Similarly, CD8^+^ T cells, B cells and T cells scores were down‐regulated in high‐LEPRE1 subgroup (Figure 3B). Besides, the marker genes of CD8^+^ T cells (Figure 3F, Table S5.3) and T inflamed cells score (Figure 3G) had a negative correlation with LEPRE1 expression. Similarly, Interferon γ and chemokine receptors pathway was downregulated in high‐LEPRE1 subgroup (Figure 3C, Table S5.2).

A large number of chemokines were negatively correlated with LEPRE1 (Figure 3D, Table S5.5). The results of the cancer immunity cycle showed that the high‐LEPRE1 subgroup had down‐regulated levels of Priming and activation and Trafficking of T cells to tumours. Furthermore, the infiltration of T cells into tumours and recognition of cancer cells by T cells were identified to be upregulated in the low‐LEPRE1 subgroup (Figure 3E). LEPRE1 was identified to be negatively correlated with ICB including PD‐1/L1, CTLA4 and TIGIT (Figure 3H, Table S5.4).

In addition, our findings were validated in IHC samples of 80 cohorts. Through the IHC spatial distribution of CD8^+^ T cells, samples were classed into deserted, excluded and inflamed phenotype (Figure 4A). The IHC scores and expression of PD‐L1 were the highest in the inflamed phenotype (Figure 4D,E). Additionally, CD8^+^T and PD‐L1 IHC scores had a negative correlation with LEPRE1 IHC scores (Figure 4B,C). Meanwhile, LEPRE1 IHC scores and expression were the lowest in the inflamed phenotype (Figure 4F,G). Finally, we found that the mRNA of LEPRE1 was highest in immune/tumour cells of deserted phenotype in ESCC (Figure 4H,I).

**FIGURE 4:**
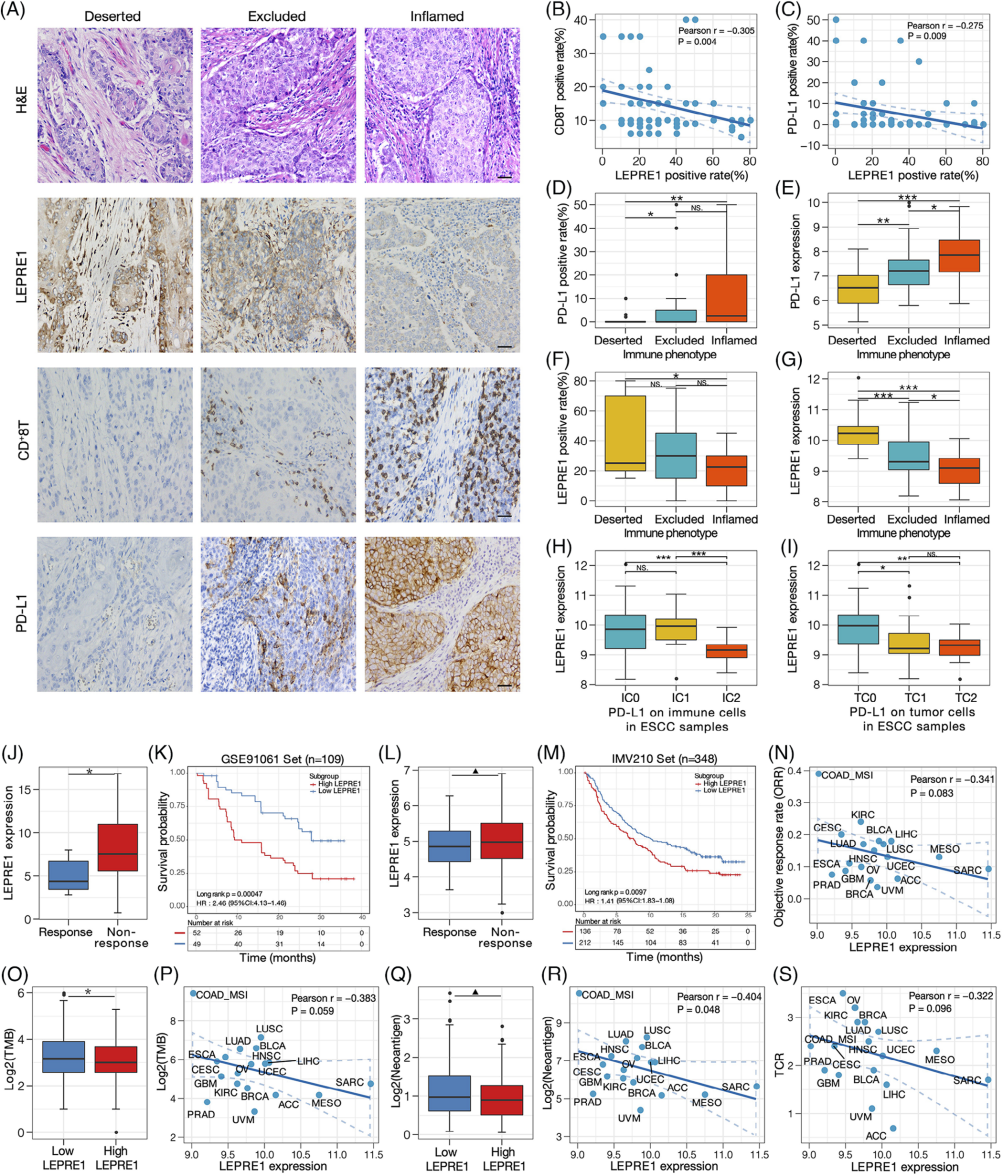
Correlations between LEPRE1 and the immune phenotype in Chinese esophageal squamous cell carcinoma (ESCC), evaluation of correlates of response to anti‐PD‐L1 therapy across cancer types in TCGA. (A) Expression of LEPRE1, CD8^+^T and PD‐L1 in Chinese ESCC cohort (*n* = 80) were detected using IHC and H&E. Representative staining images of LEPRE1, PD‐L1 and CD8^+^T in three immune phenotypes. Esophageal cancer tissues were classified into three immune phenotypes including deserted, excluded and inflamed phenotypes, based on the spatial distribution of CD8^+^ T cells. The scale bars correspond to 200 μm. (B and C) Correlation between the LEPRE1 positive rate and CD8^+^T, PD‐L1 positive rate detected using IHC in Chinese ESCC cohort. (D and E) PD‐L1 IHC score and mRNA expression in the three immune phenotypes of Chinese ESCC cohort. (F and G) The positive rate and mRNA expression of LEPRE1 in three phenotypes in Chinese ESCC. (H and I) Differences in the PD‐L1 expression on immune cells, and the PD‐L1 expression on tumour cells between high‐ and low‐LEPRE1 subgroups in Chinese ESCC. (J and L) Distribution of LEPRE1 expression in different clinical response of cancer immunotherapy in the GSE91061(*n* = 109) and IMvigor210 (*n* = 348) cohort. (K and M) Survival curves are shown for high‐ and low‐LEPRE1 expression in PD‐L1 treatment cohort. (O and Q) Differences in the TMB and neoantigen load between high‐ and low‐LEPRE1 subgroups in IMvigor210 cohort. (N, P, R and S) Pearson correlation between LEPRE1 expression and objective response rate (ORR), TMB, neoantigen load, T cell receptor (TCR) diversity with the objective response rate to anti‐PD1/PD‐L1 therapy across cancer types in TCGA. ^▲^ *p* < 0.1; *p < 0.05; **p < 0.01; ***p < 0.001. IHC, immunohistochemistry; response (PD, progressed disease; SD, stable disease); non‐response (PR, partial response; CR, complete response). Significance for survival analysis was calculated by the long‐rank test.

To explore the predictive value of LEPRE1 on clinical response to immunotherapy. The LEPRE1 mRNA was higher in patients with a progressive/stable disease than the patients with a partial/complete response to ICB (Figure 4J,L). Meanwhile, we revealed that the low‐LEPRE1 subgroup survived better than the high‐LEPRE1 subgroup in GSE91061 and IMV210 cohort (Figure 4K,M). In addition, TMB and neoantigen load were decreased in tumours with high‐LEPRE1 subgroup (Figure 4O,Q). A negative correlation among the LEPRE1 mRNA and objective response rate, TMB, neoantigen and T cell receptor (TCR) obtained from TCGA (Figure 4N,P,R,S).

Conclusively, we revealed that LEPRE1 expression correlates with tumour proliferation and metastasis in ESCC and pan‐cancers. Besides, experimental data showed that LEPRE1 promoted the proliferation and metastasis in ESCC cells. Mechanistically, LEPRE1 increased EMT and ECM by regulating the TGF‐β signaling pathway, which contributes to immune escape in ESCC and shaping a non‐flamed tumour immune landscape. Additionally, single‐cell RNA sequencing data of human esophagus tissues (GSE103239, Figure S4) showed that high LEPRE1 expression and activation of TGF‐β pathway restricted the infiltration of CD8^+^ T cells into tumour cells and inhibited immunotherapy response in ESCC.

## CONFLICT OF INTEREST STATEMENT

The manuscript describes original work and has not been submitted elsewhere for publication. All the authors listed in the manuscript declare there is no conflict of interest and have approved the manuscript that is enclosed.

## FUNDING INFORMATION

National Natural Science Foundation of China, Grant Number: 82260600; State Key Laboratory of Pathogenesis, Prevention and Treatment of High Incidence Diseases in Central Asia Fund, Grant Number: SKL‐HIDCA‐2019‐14; Key Project of Xinjiang Uygur Autonomous Region Natural Science Foundation, Grant Number: 2022D01D69; Distinguished Young Project of Natural Science Foundation of Xinjiang Uygur Autonomous Region, Grant Number: 2022D01E76; Leading Talent Project of Scientific and Technological Innovation in Tianshan Talents Training Plan of Xinjiang Uygur Autonomous Region, Grant Number: 2022TSYCLJ0031; Youth Fund of Affiliated Tumor Hospital of Xinjiang medical University, Grant Number: 2021YQ002

## ETHICS APPROVAL AND CONSENT TO PARTICIPATE

This study was approved by the Ethics Committee of the Affiliated Tumor Hospital of Xinjiang medical University (No. K‐2021025).

## DECLARATION OF COMPETING INTEREST

The manuscript describes original work and has not been submitted elsewhere for publication. All the authors listed in manuscript declare there is no conflict of interest and have approved the manuscript that is enclosed.

## Supporting information

Supporting Information

Supporting Information

Supporting Information

Supporting Information

Supporting Information

Supporting Information

## Data Availability

The raw genomic, transcriptomic data and clinical data from the Xinjiang Tumor Hospital of China have been uploaded to the Genome Sequence Archive of Beijing Institute of Genomics, Chinese Academy of Sciences (https://ngdc.cncb.ac.cn/gsa‐human) under accession number HRA000178. All relevant data will be shared on reasonable request to the corresponding authors.
