# Supplementary figures and images for: LEPRE1 shapes a non‐flamed tumour immune landscape and predicts the prognosis in esophageal squamous cell carcinoma
Source: Clin Transl Med. 2023 Nov 23;13(11):e1473. doi: 10.1002/ctm2.1473 (PMC10667621; doi:10.1002/ctm2.1473)

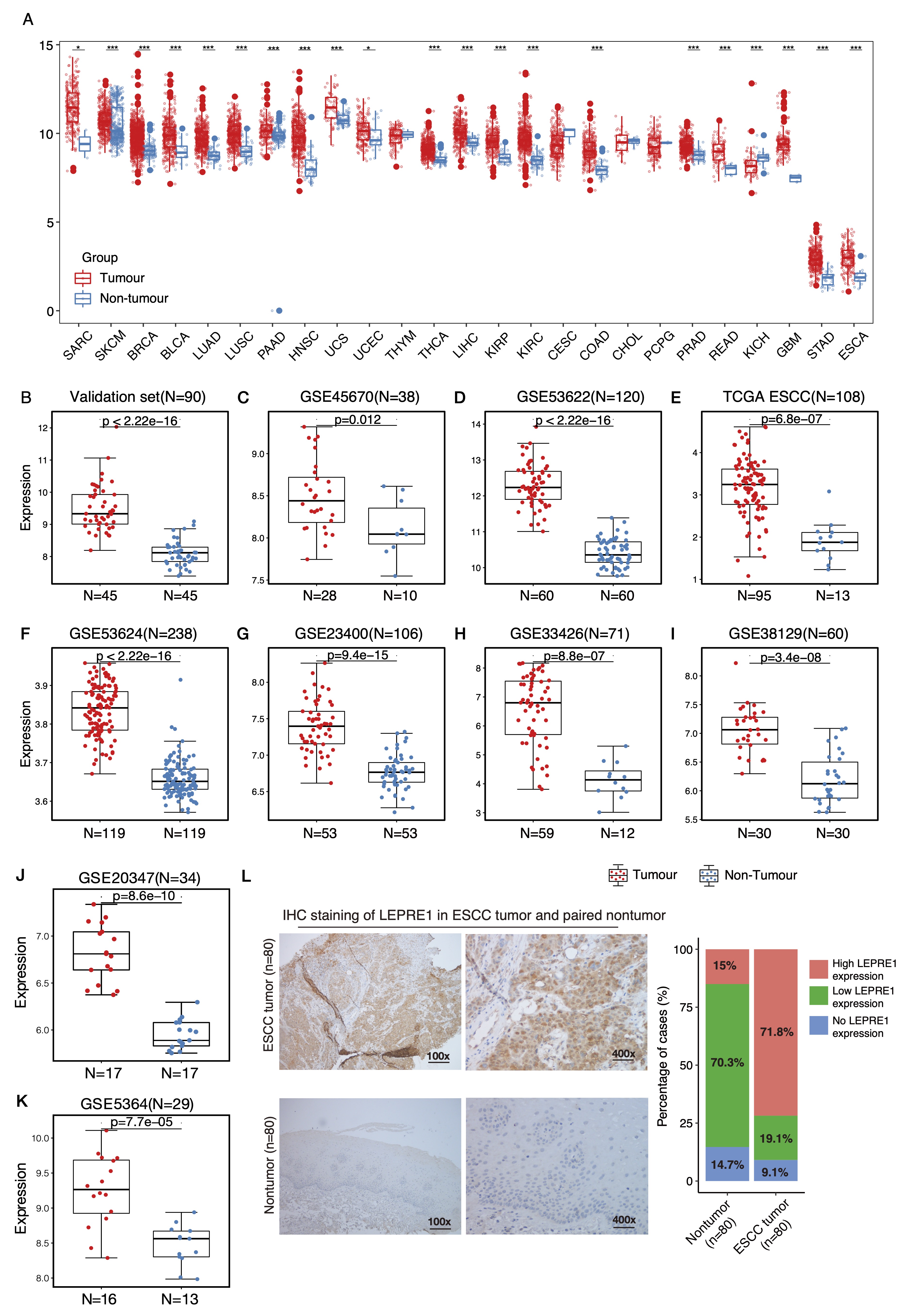

Supplement: Supplementary file 2 — Supporting Information [file CTM2-13-e1473-s002.jpg]

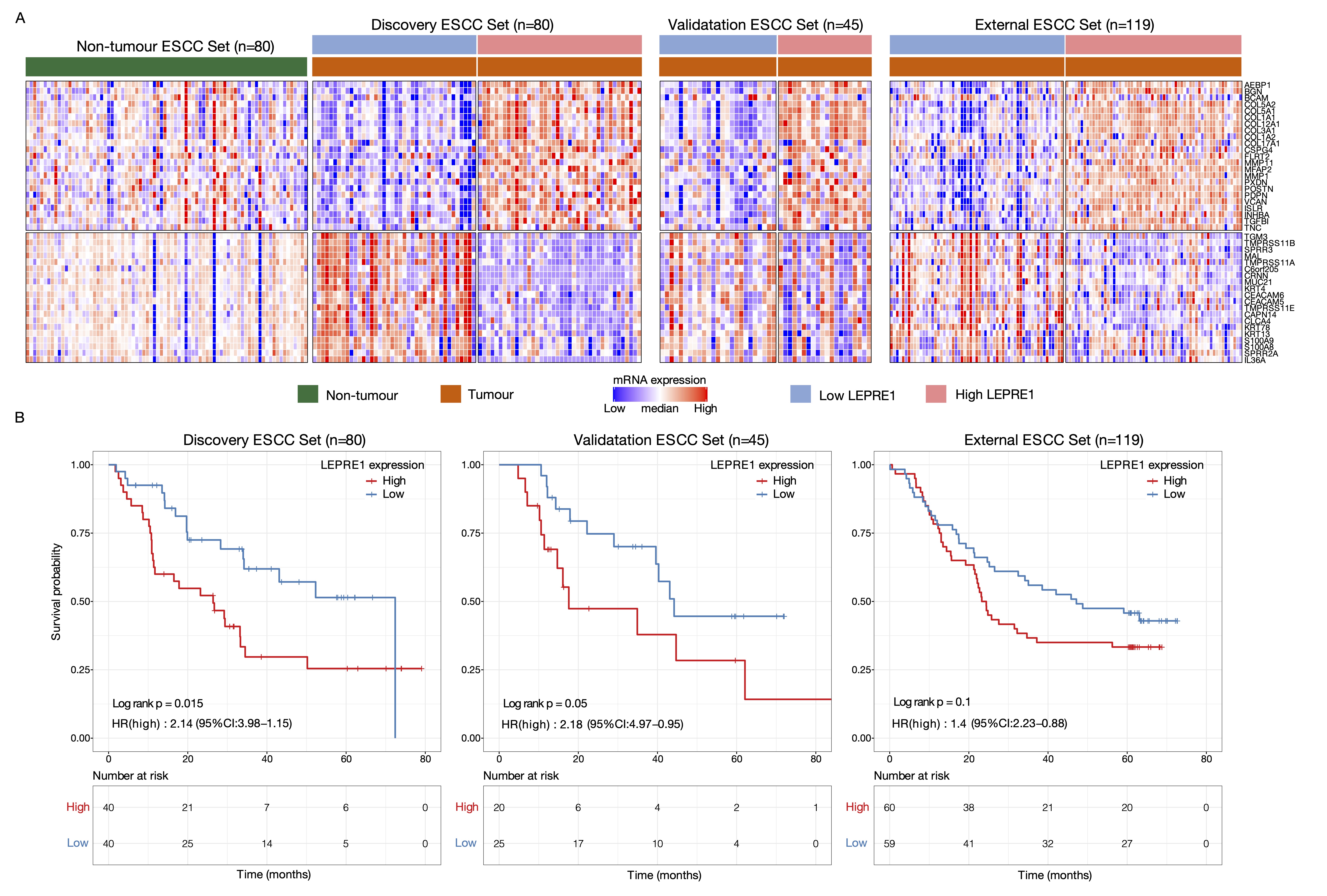

Supplement: Supplementary file 3 — Supporting Information [file CTM2-13-e1473-s001.jpg]

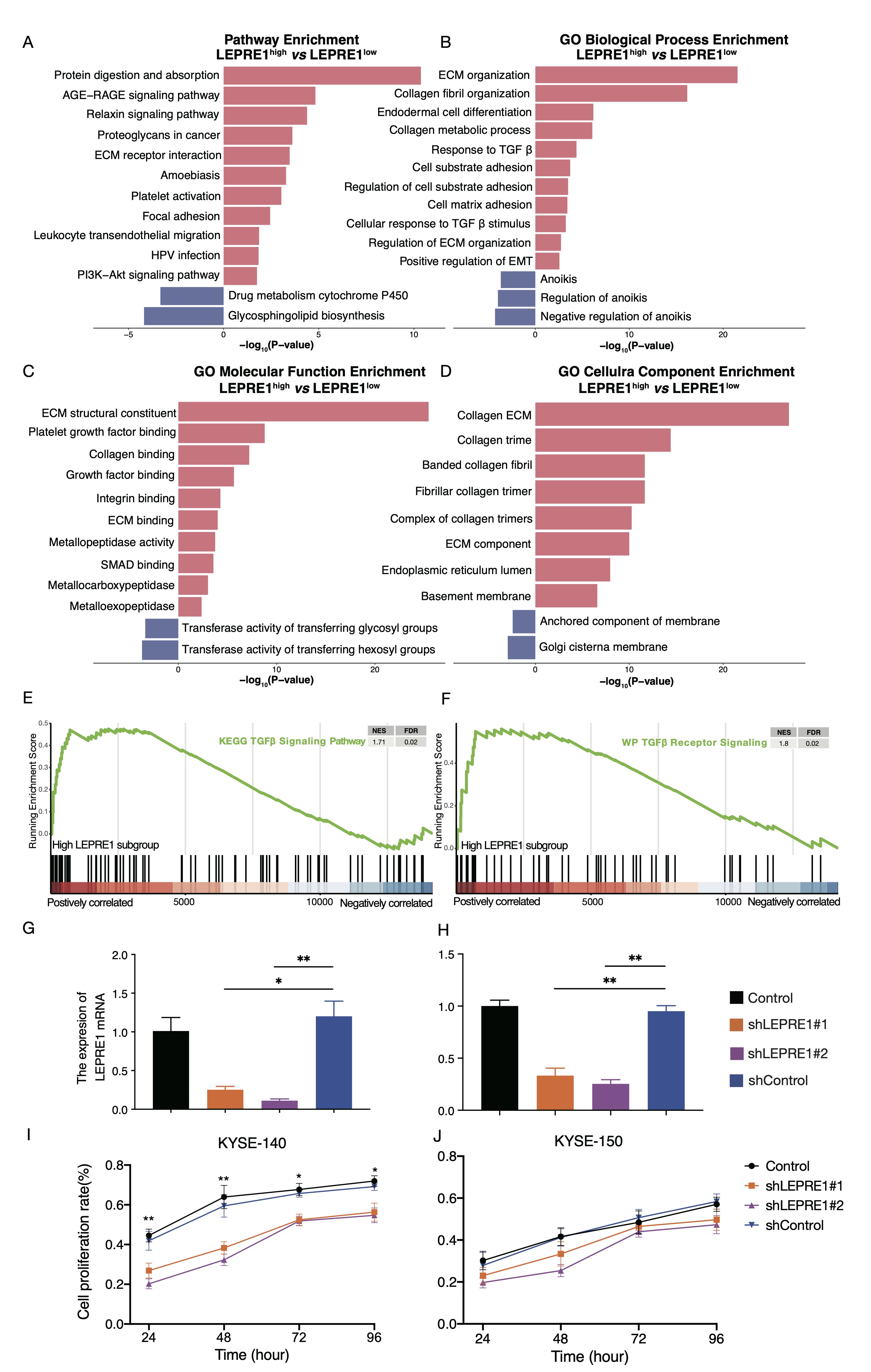

Supplement: Supplementary file 4 — Supporting Information [file CTM2-13-e1473-s005.jpg]

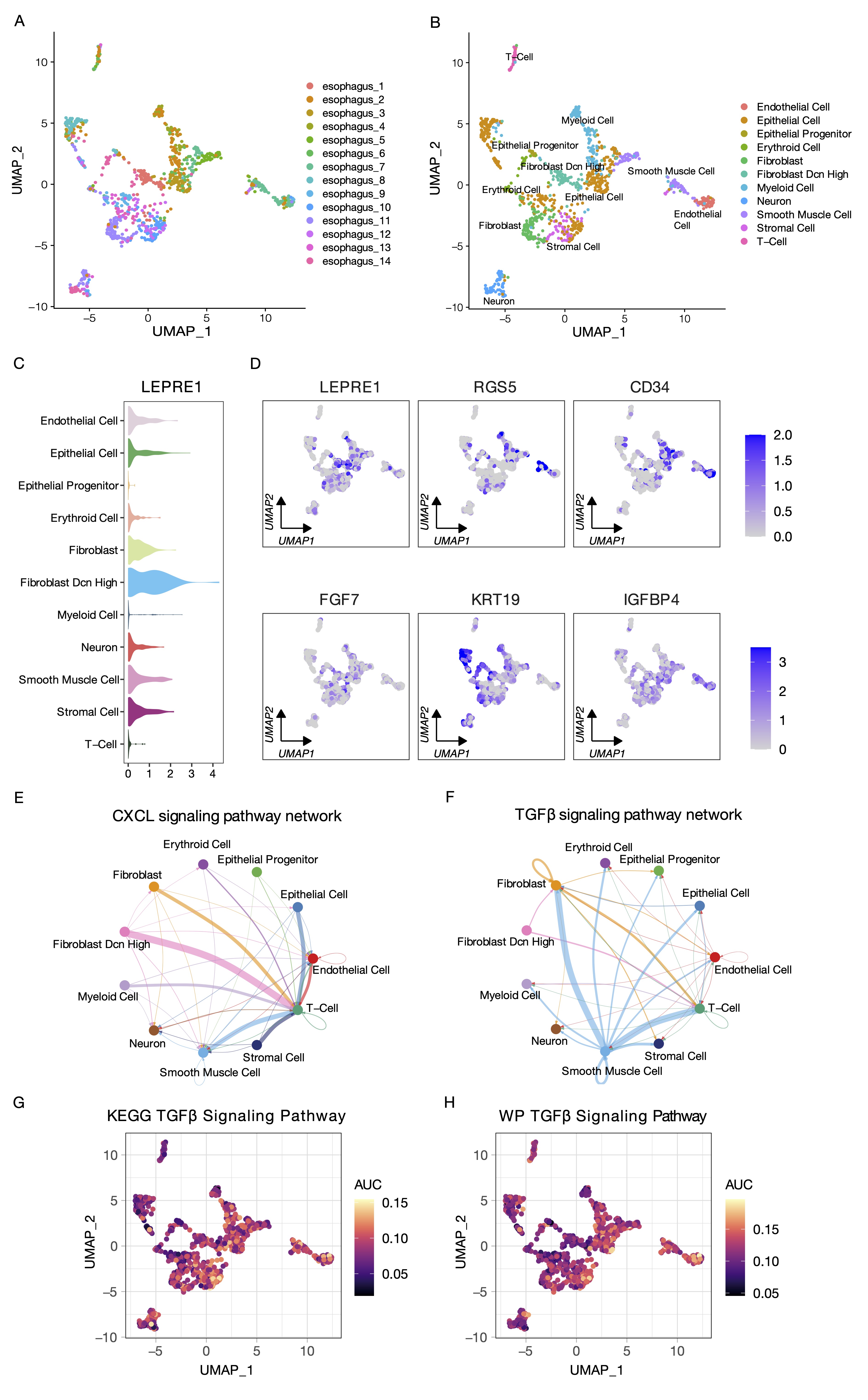

Supplement: Supplementary file 5 — Supporting Information [file CTM2-13-e1473-s004.jpg]
