# Supplementary material for: LEPRE1 shapes a non‐flamed tumour immune landscape and predicts the prognosis in esophageal squamous cell carcinoma
Source: Clin Transl Med. 2023 Nov 23;13(11):e1473. doi: 10.1002/ctm2.1473 (PMC10667621; doi:10.1002/ctm2.1473)
Supplement: Supplementary file 6 — Supporting Information [file CTM2-13-e1473-s006.docx]

**MATERIALS AND METHODS**

**1. Dataset collection**

Chinese ESCC transcriptomic data and clinical features of 80 and 45 patients respectively from the Xinjiang Tumor Hospital and somatic mutation status from whole-exome sequencing (WES) of 50 samples were obtained from BIG (HRA000178)[1]. Methylation and miRNA-lncRNA expression data were obtained from TCGA ESCC website (https://portal.gdc.ca ncer.gov/repository). The ESCC expression data and detailed clinical information of GSE53622, GSE53624, GSE45670, GSE23400, GSE33426, GSE38129, GSE20347 and GSE5364 were downloaded from the Gene Expression Omnibus (GEO) (http://www.ncbi.nlm.nih.gov/geo/). Two immunotherapy-related cohorts GSE91061 and IMV210 cohorts[2] were also downloaded. The mRNA expression data and clinical features across 33 human cancers were downloaded from TCGA (https://portal.gdc.cancer.gov/). The mRNA expression data of cancer cell lines and normal tissues were respectively downloaded from the Cancer Cell Line Encyclopedia (CCLE) (<https://portals.broadinstitute.org/ccle/about)> and GTEx (https://www.gtexportal.org/home/).

**2. Characterize the expression alteration and survival analyses of LEPRE1 mRNA in ESCC patients**

We used R package limma to analyze the differential expression genes between tumor and paired normal samples from our ESCC RNA-seq, TCGA, GEO data, and |fold change| > 1.5 and false-discovery rate (FDR) < 0.01 were defined as significant[3]. Cox model and log-rank test were used to analyze whether LEPRE1 expression was associated with the overall survival (OS) times in ESCC patients (FDR < 0.05 indicate significance). We also used a person’s chi-squared test for comparisons of ordered categorical variables to evaluate the difference of clinical relevant events, including lymph node metastases (LNM), stage, grade, subtype and survival across different cancer types, FDR < 0.05 indicate significance.

**3. Immunohistochemistry (IHC) and assay method**

IHC was performed as described previously[4]. Briefly, staining was performed using a LEPRE1 (ab4055, Abcam), CD8^+^T (ZA-0508, Zsbio) and PD-L1 (ab213524, Abcam) antibody in combination with a secondary antibody [Enzyme labelling goat anti-mouse/rabbit immunoglobulin G (IgG)] (PV-6000, Zsbio). For the IHC scoring of LEPRE1, CD8^+^T and PD-L1, we assessed the staining intensity of tumor tissue at low magnification. Samples with no staining in cancer cells were assessed score 0, weakly stained samples scored 1, samples stained with moderate intensity scored 2, and samples with a strong intensity of staining scored 3. Positive cells from randomly selected five high-magnification fields were calculated. Samples with < 25%, 25%–50%, 50%–75% and ≥ 75% positive expression were assessed score 1, 2, 3 and 4 respectively. For LEPRE1 staining, we estimated the intensity score with the positive expression value. The expressions of CD8^+^T and PD-L1 were identified only the percentage of cells with a strong intensity of membrane staining. Samples were classified into three phenotypes through the spatial distribution of CD8^+^ T cells. CD8^+^ T cells located in the tumor parenchyma were defined as “inflamed phenotype”; CD8^+^ T cells located in the stroma surrounding the tumor but not in parenchyma were named as “excluded phenotype”; and CD8^+^ T cells were neither tumor parenchyma nor stroma, characterized as “deserted phenotype”[5]. Two pathologists reviewed all slides independently.

**4. Functional and pathway enrichment analysis**

We first divided patient samples into two subgroups (high LEPRE1 expression *vs.* low LEPRE1 expression) by median expression value of LEPRE1 across 33 cancer types and 80 Chinese ESCC. Gene annotation enrichment analysis was estimated with the R package ClusterProfiler[6]. We then identified the up-regulated and down-regulated pathways and Gene Ontology (GO) terms between high- and low-LEPRE1 Chinese ESCC subgroups. Additionally, we also assessed the enrichment in comparison of the two groups across 33 cancer types using a gene set enrichment analysis (GSEA) 4.2.3 (<https://www.gsea-msigdb.org/gsea/index.jsp)>. The gene sets were downloaded from the Molecular Signatures Database (MSigDB) v.5.2 (<http://software.broadinstitute.org/gsea/msigdb/index.jsp)>, FDR were calculated using Benjamini-Hochberg correction[7]. FDR less than 0.05 were considered significant.

**5. Single-sample gene-set enrichment analysis of tumor immune processes**

A single sample gene set enrichment analysis (ssGSEA) was applied to evaluate the tumor-immune microenvironment and pathway activation status in each sample. First, gene signature scores with respect to the immune cells and pathways across 33 cancers from TCGA and Chinese ESCC (n=80) were calculated for each sample using the ssGSEA algorithm by gene set variation analysis (GSVA) package[8]. And then, 22 ICIs with therapeutic potential were collected as studied previously[9]. Finally, spearman analysis was used to assess the relationship between LEPRE1 and immune cells, ICIs. The correlation between LEPRE1 and T cell-inflamed score was analyzed to confirm the role of LEPRE1 in modulating cancer immunity and predicting the clinical response of immune checkpoints in ESCC. Additionally, immune cells, pathways and T cell-inflamed gene sets derived from MSigDB (V.6.2) were detailed in the Table S6.

**6. Cell culture and gene Knockdown in ESCC Cells**

Human ESCC cell lines KYSE150 and KYSE140 were purchased from iCell Bioscience Inc (Shanghai China) and Otwo Biotech Inc (Shenzhen China). These cell lines were cultured in RPMI 1640 medium with 10% fetal bovine serum (FBS). To confirm that LEPRE1-regulators confer the proliferation, invasion and metastasis for ESCC cells, the KYSE140 & KYSE150 cells with stable knockdown LEPRE1 were cultivated by shRNA lentiviral plasmid construct infection. The pSLentivirus-U6-shRNA expression vector was used to construct shRNA expressing plasmids. The lentiviruses encoding shRNA sequences for LEPRE1 (sh#1: CCAGGCCATCACAGATCATTA; sh#2: GGCAGAGAGGAAGGATGATAG) and control shRNA were produced according to the manufacturer’s instructions (OBIO Technology Corp., Ltd, Shanghai China). KYSE140 and KYSE150 cells were infected with these lentiviruses, and the stable knockdown cells were obtained for the following 1 μg/ml puromycin selection for one week.

**7. Cell viability and migration or invasion assays**

Cell Counting Kit-8 (CCK-8) (APExBIO, K1018) was used for the cell viability. Briefly, the knockdown cells were seeded into 96-well plates for 2 days. CCK8 was added to each well according to the manufacturer’s instructions. The absorbance was performed at 450 nm. The data was visualized using R software. Invasion assays were measured in 24-well chambers (Corning) coated with Matrigel (Corning). Cells (2 × 10^5^) in serum-free medium were added to the coated chamber and incubated for 18 or 24 h before fixed with methanol and stained with 0.5% crystal violet. Migration assays were analyzed in a similar experimental procedure but without coating the filters with Matrigel.

**8. Quantitative real-time PCR analysis**

Total RNA of human ESCC Cell lines was extracted with TRIzol (Invitrogen) and the reverse transcription was measured using PrimeScript TM RT reagent kit (Thermo Fisher scientific). Quantitative real-time PCR (qRT-PCR) was analyzed in triplicate using 2×Taq PCR MasterMix Ⅱ (TIANGEN). Primer sequences for LEPRE1 and GAPDH were as follows: LEPRE1(forward primer: 5’-GTG ATG GAC GGC GTA ATC TCT GAC-3’; reverse primer:5’-GAG GGC TTT GAA GAC AGT GAC ACC-3’); GAPDH (forward primer: 5’-GTA TGA CAA CAG CCT CAA GAT-3’; reverse primer:5’-GTC CTT CCA CGA TAC CAA AG-3’) (Sangon Biotech).

**9. Western blot analysis**

Proteins of Human ESCC Cell lines were extracted by RIPA lysis buffer (Solarbio, R0010) containing PMSF. In general, lysate containing 10-20μg of protein was separated on SDS-PAGE and transferred to PVDF membranes (Biosharp). Antibodies against E-cadherin (BS-10009R) and N-cadherin (BS-1172R) were from Bioss while antibodies against Vimentin (E-AB-10967) were from Elabscience. Images were acquired on a bio-rid confocal system. The signal was calculated with a SuperSignal^TM^ West Pico PLUS (Thermo Scientific, 34579) analyzed through the Tanon-2500.

**10. Single-cell RNA sequencing data analysis**

Single-cell RNA sequencing data of human esophagus tissues were downloaded from GSE103239[10], and then we performed Seurat (v4.2.0) standardized workflow. Besides, CellChat was used to inferred cell-cell communications[11]. Cells of human esophagus tissues were annotated by canonical markers. AUCell is used to demonstrate cells with active gene regulatory networks in single-cell RNA sequencing data of human esophagus tissues[12].

**11. Statistical analysis[13]**

Correlations between variables were explored using Pearson or Spearman coefficients. Continuous variables fitting a normal distribution between binary groups were compared by t-test; Otherwise, the Wilcoxon (for two groups) or Kruskal-Wallis (for more than two groups) test was used. Categorical variables were compared using the chi-squared test or Fisher’s exact test. Kaplan–Meier survival analysis and Cox regression model were used to analyze the prognostic value of LEPRE1 by the packages ‘survival’ and ‘survminer’, and the log-rank test was applied to determine statistical significance. The level of significance was set at P < 0.05, and all statistical tests were two-sided. All statistical data analyses were performed by R software, version 4.1.0.

**References**

1. Liu M, An H, Zhang Y, Sun W, Cheng S, Wang R, et al. Molecular analysis of Chinese oesophageal squamous cell carcinoma identifies novel subtypes associated with distinct clinical outcomes. EBioMedicine. 2020; 57: 102831.

2. Mariathasan S, Turley SJ, Nickles D, Castiglioni A, Yuen K, Wang Y, et al. TGFβ attenuates tumour response to PD-L1 blockade by contributing to exclusion of T cells. Nature. 2018; 554: 544-8.

3. Ritchie ME, Phipson B, Wu D, Hu Y, Law CW, Shi W, et al. limma powers differential expression analyses for RNA-sequencing and microarray studies. Nucleic Acids Res. 2015; 43: e47.

4. Yu C, Chen K, Zheng H, Guo X, Jia W, Li M, et al. Overexpression of astrocyte elevated gene-1 (AEG-1) is associated with esophageal squamous cell carcinoma (ESCC) progression and pathogenesis. Carcinogenesis. 2009; 30: 894-901.

5. Desbois M, Udyavar AR, Ryner L, Kozlowski C, Guan Y, Durrbaum M, et al. Integrated digital pathology and transcriptome analysis identifies molecular mediators of T-cell exclusion in ovarian cancer. Nat Commun. 2020; 11: 5583.

6. Yu G, Wang LG, Han Y, He QY. clusterProfiler: an R package for comparing biological themes among gene clusters. OMICS. 2012; 16: 284-7.

7. Storey JD. A direct approach to false discovery rates. Journal of the Royal Statistical Society: Series B (Statistical Methodology). 2002; 64: 479-98.

8. Hänzelmann S, Castelo R, Guinney J. GSVA: gene set variation analysis for microarray and RNA-seq data. BMC Bioinformatics. 2013; 14: 7.

9. Auslander N, Zhang G, Lee JS, Frederick DT, Miao B, Moll T, et al. Robust prediction of response to immune checkpoint blockade therapy in metastatic melanoma. Nat Med. 2018; 24: 1545-9.

10. Gao S, Yan L, Wang R, Li J, Yong J, Zhou X, et al. Tracing the temporal-spatial transcriptome landscapes of the human fetal digestive tract using single-cell RNA-sequencing. Nat Cell Biol. 2018; 20: 721-34.

11. Jin S, Guerrero-Juarez CF, Zhang L, Chang I, Ramos R, Kuan CH, et al. Inference and analysis of cell-cell communication using CellChat. Nat Commun. 2021; 12: 1088.

12. Aibar S, Gonzalez-Blas CB, Moerman T, Huynh-Thu VA, Imrichova H, Hulselmans G, et al. SCENIC: single-cell regulatory network inference and clustering. Nat Methods. 2017; 14: 1083-6.

13. Sebastiao YV, St Peter SD. An overview of commonly used statistical methods in clinical research. Semin Pediatr Surg. 2018; 27: 367-74.
